# Liq. Magnesiæ—A Remedy for Cardialgia (Heartburn.)

**Published:** 1872-02

**Authors:** 


### LIQ. MAGNESIÆ BISULPHITIS—A REMEDY FOR CARDIALGIA (HEARTBURN.)

Some time ago a physician asked me the question, "Do the bisulphites prevent the butyric acid fermentation?" In order to give him an accurate answer, I promised to try two experiments. This I did. First, I proceeded to make butyric acid by fermentation of a mixture of chalk, cheese, and honey and water, and allowed the mixture to stand for four days. Secondly, in another vessel I proceeded in the same manner, using the same ingredients, with an addition of bisulphite of lime; set it aside for four days with the first, keeping them at a temperature of eighty degrees Fahrenheit; after which I subjected each to distillation with a little H Cl.* From the *first* I recovered a considerable amount of butyric acid, but from the second (containing bisulphite) I did not recover a trace. This at once proves that the bisulphites do prevent butyric acid fermentation. Now, the object in ascertaining the fact was, that a suitable remedy for heartburn might be discovered, as, according to Dr. Leared, this common com-

*For the first two days lactic acid is formed, which combines with the lime, but at the expiration of four days, the lactate of lime is replaced by butyrate of lime, which, on being distilled with dilute hydrochloric acid, and the distillate treated with calcium chloride, is dried into two strata, the upper being butyric acid.

plaint is due to the presence of butyric acid in the stomach. "On considering the taste," says that gentleman, "experienced, as well as the conditions under which heartburn comes on, it seemed to me that the cause of it was the presence of butyric acid;" and from many experiments performed by that gentleman on himself and others by means of the pure acid, symptoms were produced in every respect similar to the complaint itself, so that there can be little doubt but that his theory is a correct one. The very fact that alkalies gives relief proves its cause to be from an acid. When the stomach is overtaxed, and in certain weak conditions of digestion, fermentation takes place; butyric acid is set free from the food—*i. e.*, it is formed out of its own elements, if the food be of a starchy nature; and, according to Leared "On Imperfect Digestion," page 249, "the acid, by being in excess, but not pure (or it would be soluble), rises to the surface of the contents of the stomach, when it combines with melted fats (for which it appears to have a strong affinity); the acrid mixture, on being presented to the cardiac orifice by the motions of the stomach, is instinctively rejected into the œsophagus, and, by the reversal of its proper movement, transmitted to the mouth, accompanied by the sensations of heartburn." Now, as bisulphites have the power of preventing this fermentation, they are well worthy the attention of the profession; but the great drawback is that the chief bisulphite manufactures are those of lime, soda and potash, these being objectionable, as they tend to injure the coats of the stomach. To remedy this failing, the thought at once suggested itself to me, that a bisulphite of magnesia might be prepared; and magnesia being free from these objections, it may prove a valuable remedy, and is worth notice. I have not seen or heard anything of the preparation previous to my making it. I therefore give a brief outline of the process I adopt, and hope to enter more fully into the subject at a future time. I first treat magnesia carbonate with B. B. sulphurous acid, which, on evaporation, yields magnesia sulphite, $Mg\ SO_3$, which is not very soluble in distilled water. I then mix the sulphite of magnesia thus formed with distilled water, in the proportion of sixteen grains to ℥j, and pass into it sulphurous anhydride until a clear solution is obtained. The result is a solution of magnesia bisulphite.

The dose may be one table-spoonful, containing about nine grains of the salt; its action is a mild aperient antiseptic, preventing butyric fermentation in the stomach, etc. I have tried it on myself and on two other gentlemen, and, as far as I can judge, it has the desired effect.—*Geo. Archbold, D.SC., in Pharm. Journal and Transactions, December* 23, 1871.

